# Obstacles and alternative options for cardiac rehabilitation in Nanjing, China: an exploratory study

**DOI:** 10.1186/1471-2261-14-20

**Published:** 2014-02-17

**Authors:** Hong Jin, Qin Wei, Long Chen, Qin Sun, Yun Zhang, Juan Wu, Genshan Ma, Naifeng Liu

**Affiliations:** 1 Department of Cardiology, Zhongda Hospital, Medical School of Southeast University, 210009 Nanjing, Jiangsu, China

**Keywords:** Coronary heart disease, Secondary prevention, Cardiac rehabilitation, Health education

## Abstract

**Background:**

Coronary heart disease (CHD) is a major cause of morbidity and mortality, and cardiac rehabilitation (CR) is still not well developed in mainland China. The objective of this study is to investigate the barriers associated with those seeking cardiac rehabilitation (CR) and to explore appropriate secondary prevention modalities tailored to the needs of Chinese patients with coronary heart disease (CHD).

**Methods:**

A consecutive series of eligible patients was recruited from the cardiac department of a teaching hospital in Nanjing, located in southeast China. Structured face-to-face interviews were conducted with 328 patients prior to hospital discharge. Patient preferences for seeking an outpatient CR program or an alternative outpatient self-choice, minimal-cost educational program were evaluated. Socio-demographic characteristics and clinical data were assessed. Additionally, patients were asked to provide the reasons affecting their choice.

**Results:**

Overall, only 14.3% patients preferred the standard CR program. Factors associated with non-participating were female gender (odds ratios [ORs], 6.05, 95% CI, 1.30-28.19), older age (ORs, 1.11, 95% CI, 1.04-1.19, per year), less education (ORs, 8.13, 95% CI, 2.83-23.38), low income (ORs, 3.26, 95% CI, 1.24-8.54), and having either basic medical care or a lack of health insurance (ORs, 10.01, 95% CI, 3.90-25.68). The most common reason for refusing to participate in CR was that patients could not afford it. Of the remaining patients, 65.8% patients chose self-choice educational programs, especially for female (ORs, 5.84, 95% CI, 2.67-12.79), older (ORs, 1.06, 95% CI, 1.02-1.11, per year), and low income (ORs, 2.14, 95% CI, 1.12-4.10) patients. The main reasons for their preferences were their desires for more information about disease and risk factors, the low cost, feasibility, and saving time.

**Conclusions:**

Multiple barriers, which may occur at the patient, health system, and societal levels, have prevented eligible patients from participating in CR programs. Self-choice educational programs, an alternative model incorporating more information, would strongly meet the needs of most patients. A feasible delivery format for secondary prevention should be provided for all CHD patients.

## Background

Coronary heart disease (CHD) is a major cause of morbidity and mortality in China, despite advances in medical treatment [1-3]. Urbanization, industrialization, and the aging of the population have resulted in a rapid and significant increase in the prevalence and incidence of CHD in the past decades [1]. Uncontrolled risk factors, unhealthy lifestyles, and lack of knowledge about the disease have resulted in poor management of CHD among Chinese patients [4,5].

The high mortality and morbidity associated with CHD has resulted in calls for the universal provision of rehabilitative and preventive measures for all patients with this disease [6]. During the past decades, many studies conducted in Western countries have demonstrated that cardiac rehabilitation (CR) can significantly reduce cardiac risk and symptoms, improve functional capacity, enhance psychological well-being and reduce the risk of further cardiac events [7-9]. However, even in many developed countries, participation rate in hospital-based CR programs are reported to be low, particularly for women [10,11], the elderly [12,13], patients at a lower socioeconomic status [14], patients with less education [15] and patients lacking insurance [16]. Many attempts have been made by rehabilitation centers to offer choices in the format of the program, in an effort to encourage participation. These alternatives have included individual consultations with health professionals and self-education [17], as well as modular approaches [18], group counseling programs [19], and home-based CR programs [20]. Randomized trials studying the effects of these alternative CR models have demonstrated beneficial outcomes for patients participating in such programs after cardiac events [18-20].

In developed Western countries, CR is a rapidly developing area on health care. However, in mainland China, the concept of CR is relatively new and has received little attention [21]. In contrast to the high numbers of emergency percutaneous coronary intervention (PCI) performed, CR is still in its infancy, and CR services are rarely found in most parts of the country [5]. This fact is surprising, considering the size of the country and its population. A lack of priority, limited health care resources and scarce rehabilitation facilities are believed to pose major challenges to the development and implementation of CR programs in mainland China [5]. To date, there is no standard design for CR programs in mainland China. In searching for barriers to CR and potential solutions to the CR gap, we firstly introduced a standard CR model based on a 3-month outpatient program for patients following acute coronary syndrome (ACS) [22] in our center. Secondly, if patients did not agree to participate in the standard CR program, we offered an alternative self-choice educational program for increasing knowledge about heart disease and stimulating lifestyle changes.

Therefore, the aims of this study were: 1) to identify factors and reasons for non-participating in the standard CR program; 2) to investigate whether patients preferred an alternative self-choice educational program model for getting information; 3) to further evaluate predictors for attending the alternative self-choice educational program. Finally, 4) the desire for specific information regarding heart disease and preventive therapies were also examined.

## Methods

### Patient population

**Figure 1:**
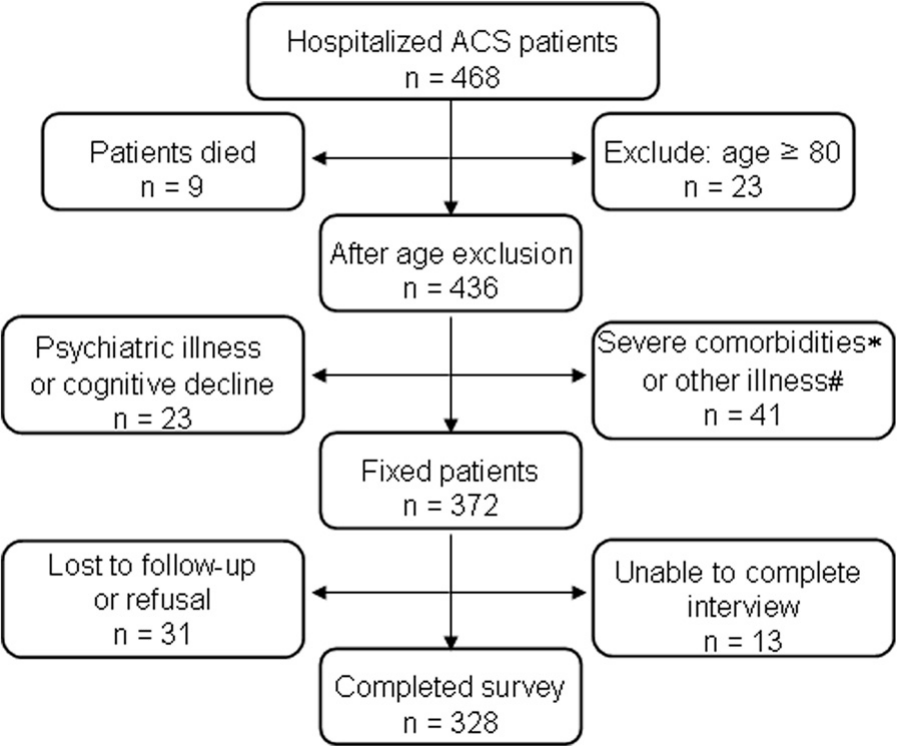
**Flowchart for recruitment.** ACS, acute coronary syndrome; ^*^Includes significant cardiac comorbidities and advanced cancer; ^#^Illness that caused patients to feel unwell and precluded participation in the study (e.g., such as a transient viral or bacterial infection).

A consecutive series of patients with ACS between September 2010 and March 2012 were recruited from a cardiac center of teaching hospital with approximately 1500 beds, located in Nanjing, a provincial capital with 9 million populations. Patients were excluded if they met any of the following criteria: were over 80 years of age or had severe comorbidities, psychiatric illness or cognitive decline that impaired their ability to complete the study’s measures. A total of 468 patients were admitted to the hospital for ACS during the study period. After excluding patients based on the criteria above, there were 372 eligible patients who initially consented to participate in the study. However, 31 patients failed to follow up, and 13 patients were unable to complete interview, leaving a population of 328 patients (Figure 1). All participants gave full written informed consent, and the study was approved by the Committee of Clinical Investigation of Southeast University School of Medicine.

Data collected from the medical records included demographic information, clinical history of chronic diseases, comorbidities, cardiac diagnoses (ST segment elevated myocardial infarction (STEMI), non-ST segment elevated myocardial infarction (NSTEMI), and unstable angina), medications, and record of whether invasive procedures were performed during hospitalization (PCI, or coronary artery bypass graft (CABG)).

### Design

While patients were still in the hospital following their PCI, CABG, or continued medical treatment procedure, they were approached by a nurse from the CR team to check for eligibility and for recruitment in the study. After the acute care period, all patients were approached by a nurse from the CR team regarding participation in a standard outpatient CR course. The proposed outpatient CR program was a 12 weeks plan that included moderate intensity endurance training and education about the disease and risk factors. The endurance training was administered for 45 minutes per day for 3 times a week at a target aerobic intensity of 50% to 70% heart rate reserve. In addition, all exercise training group patients received individual counseling on exercise prescription, secondary prevention, and daily activities by a physician and a nurse over a period of 12 weeks. Patients would need to pay for the CR program at their own expense because basic medical insurance did not cover the costs of the program*.* If patients declined to attend the standard CR program, they were offered an alternative format of education regarding secondary prevention-a self-choice, minimal cost educational program, which was promoted by local enthusiasts (e.g., cardiologists (H.J.), nurses (Q.S.) or physiotherapists (Y.Z.)) who perceived a therapeutic gap and filled it, often by “borrowing” time from other professions required for this multidisciplinary activity. Patients were encouraged to attend education classes, which were held once weekly with lectures given by physicians, nurses, dieticians, and pharmacists on cardiac disease, secondary prevention, management, diet, smoking cessation, and medication.

Face-to-face interviews were conducted with 372 patients prior to hospital discharge. These interviews were approximately 20 minutes and were administered in a location convenient to the patient (e.g., bedside). Of the 372 patients who were interviewed at baseline (i.e., prior to discharge), complete data regarding all secondary prevention format preferences were available in 328 cases. The data regarding patient’ attitudes and beliefs about CR programs, as well as their preference for a self-choice educational program, were collected using a structured questionnaire. Researchers were registered nurses with graduate degrees and specialized expertise in cardiac care.

Participants completed both the face-to-face interviews and the structured questionnaire independently prior to discharge. The questionnaire surveyed the following: the first section included patient baseline characteristics such as age, gender, marital status, educational level, employment status, health insurance status, and income bracket prior to the acute event. The second section included attitudes about participation in a standard CR program. First, the researchers advised patients about the purposes, benefits, and costs associated with the CR program. Then, patients were asked if they would like to participate in a CR program. All patients responded ‘Yes’ or ‘No’. Patients who did not want to attend were asked about their reasons. To perform this assessment, they were asked, ‘Why did you decline to join the CR program?’ Patients were prompted with possible options, which included unaffordability, transportation issues, work or time conflicts, health problems, self-exercise (their desire to exercise independently of the program), skepticism towards the benefits of rehabilitation, and lack of family support, among others and were asked to choose their reasons.

The third section was explored within the group who did not agree to join a CR program. Patients were asked if they would like to attend a self-choice educational program to obtain more information about heart disease and lifestyle. Among patients who agreed to attend a self-choice educational program, we further assessed the reasons for joining the educational program, such as receiving more information about disease and risk factors, low cost, feasibility, saving time, among others. Additionally, these patients were asked to choose the specific information they desired, namely, knowledge about the disease, physical activity, diet, medication, stress management, modifying risk factors, career advice, and lifestyle changes, among others.

Additionally, before hospital discharge, all study patients were asked to complete a questionnaire, the Chinese Mandarin versions of the Short Form 36-Item (SF-36) Health Survey [23], which was used to assess the health status. The SF-36 is a widely used generic instrument consisting of 36 items. It yields 8 subscale profiles, including physical and mental health summary measurements. A high score reflects good health status. After 12 weeks from hospital discharge, SF-36 questionnaires were repeated in all study patients, including those who did not participate in CR or self-choice educational program.

Attendance at standard CR or a self-choice educational program was confirmed by a telephone survey 3 months after the initial interview, with attendance defined as having attended at least one CR or self-choice educational program session.

### Statistical analysis

Patients were divided into three groups according to whether they agreed to attend the CR program, the self-choice educational program, or refused to attend both programs. All data are expressed as the means ± standard deviations for normally distributed data and as the medians (interquartile range) for skewed continuous variables. Comparisons of continuous variables among the three groups were performed using a one way analysis of variance (ANOVA) or Kruskal-Wallis test. The Chi-square test was used to compare categorical variables across groups. A multivariable logistic regression analysis was performed to indentify factors associated with non-participating in CR, defined as refusal to participate in CR prior to hospital discharge, or attendance in a self-choice educational program. Significant univariate predictors were included in multivariate logistic regression analyses. We included 5 covariates in the model: age, gender, education, insurance, and income. Statistical analyses were performed using SPSS software 16.0 (SPSS Inc., Chicago, USA). All tests were two-sided, and a p-value of less than 0.05 was considered significant.

## Results

Overall, the mean age of the cohort was 63.0 ± 7.6 years, and 26.2% were women. A comparison of the socioeconomic and clinical characteristics of patients among three groups is shown in Table 1.

**Table 1:** Baseline characteristics of study patients

**Characteristics**	**Refusal to attend CR (n = 281)**		**Agreed to attend CR (n = 47)**	**p value**
	**Agree to attend EP (n = 185)**	**Refusal to attend EP (n = 96)**		
Age, years	65.5 ± 6.4	61.3 ± 8.3	56.7 ± 5.6	<0.001
Gender, female	75 (40.5)	9 (9.4)	2 (4.3)	<0.001
Married	181 (97.8)	94 (97.9)	44 (93.6)	0.256
Education: Junior high school or lower	125 (67.6)	59 (61.5)	5 (10.6)	<0.001
Employed	92 (49.7)	50 (52.1)	29 (61.7)	0.341
Current smoker	71 (38.4)	38 (39.6)	20 (42.6)	0.870
Health insurance status				
Basic medical care or lack of health insurance	128 (69.2)	68 (70.8)	8 (17.0)	<0.001
Free medical care or other commercial health insurance	59 (31.9)	26 (27.1)	39 (83.0)	
Income: (Chinese Yuan/month)				
Low (≤3,000)	103 (55.7)	26 (27.1)	9 (19.1)	<0.001
Medium (3,000 - 5,000)	62 (33.5)	47 (49.0)	10 (21.3)	
High (≥5,000)	20 (10.8)	23 (24.0)	28 (59.6)	
BMI (kg/m^2^)	24.3 ± 2.4	24.2 ± 2.3	23.8 ± 2.1	0.443
Medical diagnosis:				
STEMI	65 (35.1)	35 (36.5)	22 (46.8)	0.534
NSTEMI	71 (38.4)	35 (36.5)	12 (25.5)	
Unstable angina	49 (26.5)	26 (27.1)	13 (27.7)	
No. of diseased vessels	1 (1 - 2)	1 (1 - 2)	1 (1-2)	0.847
EF	50 (45 - 55)	50 (45 - 55)	50 (45 - 57)	0.740
Management strategies				
PCI	163 (88.1)	86 (89.6)	37 (78.7)	0.161
CABG	9 (4.9)	4 (4.2)	5 (10.6)	0.239
Duration of hospitalization (days)	6 (5 - 7)	6 (5 - 7)	6 (5 - 8)	0.351
Previous heart disease	51 (27.6)	21 (21.9)	16 (34.0)	0.287
Co-morbidity:				
Diabetes	60 (32.4)	22 (22.9)	15 (31.9)	0.235
Hypertension	87 (47.0)	34 (35.4)	24 (51.1)	0.105

Data are represented as the means ± SD or the median (interquartile) or number (%) of patients; SD indicates standard deviation; CR, cardiac rehabilitation; EP, educational program; BMI, body mass index; STEMI, ST segment elevated myocardial infarction; NSTEMI, non-ST segment elevated myocardial infarction; EF, ejection fraction as assessed by echocardiography; PCI, percutaneous coronary intervention; CABG, coronary artery bypass graft. P value represents statistical differences among three groups.

When compared to patients who did not agree to participate in CR, those who preferred CR were younger and more likely to be male (p < 0.001). Low income and less educated patients were more likely to prefer the educational program compared to the patients who declined the CR or educational programs (p < 0.001). Patients with basic medical care or without health insurance were less likely to prefer CR as compared to those who agreed to participate in a CR program (p < 0.001). There were no differences across groups in terms of BMI, medical diagnosis, number of diseased vessels, management strategies (PCI, CABG, or continued medical treatment), length of hospital stay, or ejection fraction as evaluated by echocardiography. In the multivariable model, factors that were independently associated with non-participation in CR included female gender, older age, basic medical care or lack of insurance, low income, and poor education (Table 2).

**Table 2:** Multivariate logistic regression model for factors associated with non-participating in cardiac rehabilitation (CR) (n = 328)

	**Adjusted ORs**	**95% CI Lower - Upper**	**p value**
Age (per year)	1.11	1.04 - 1.19	0.002
Gender			
Male	1.00	Reference group	
Female	6.05	1.30 - 28.19	0.022
Education			
> Junior high school	1.00	Reference group	
≤ Junior high school	8.13	2.83 - 23.38	< 0.001
Health Insurance			
Free or commercial	1.00	Reference group	
Basic medical care or none	10.01	3.90 - 25.68	< 0.001
Income (Chinese Yuan/month)			
Medium/high (> 3,000)	1.00	Reference group	
Low-income (≤ 3,000)	3.26	1.24 - 8.54	0.016

ORs, odds ratios; CI, confidence interval; Dependent variable, patients refused to participate in CR: non-participating = 1, participation = 0.

Reasons given by patients who refused to participate in a CR program are presented in Table 3. Patients were asked to indicate the most important reason. The most common reason for refusing to attend CR was unaffordability. Other reasons included conflicts with work or insufficient time to attend, poor health (chronic dialysis, peripheral vascular disease, trait anxiety, depression, chronic obstructive pulmonary disease*,* stroke, arthritis, and other disability/impairment), transportation problems, and lack of support from family members.

**Table 3:** Reasons for patients’ refusal to participate in cardiac rehabilitation (CR)

Reason*	**Patients (n = 281), n (%)**
Unaffordability (Cannot afford CR)	172 (61.2)
Work or time conflicts	43 (15.3)
Difficulty with commute to rehabilitation center	22 (7.8)
Health problems	15 (5.3)
Self-exercise	12 (4.3)
Family members did not support CR	11 (3.9)
Others	6 (2.1)
Considered CR to be non-essential or reluctant to join	4
Responsibility to domestic duties	2

*Patients could give only one reason.

Among patients who declined to participate in the CR program, 185 patients (65.8%) preferred an alternative educational program. The multiple regression analysis of predictors for the participation in the educational program is summarized in Table 4. Female, older, and low-income patients were more likely to attend the educational program. Unlike predictors for the participation in CR, insurance status and education level was not associated with participation rates in the alternative educational program.

**Table 4:** Multivariate logistic regression model for factors associated with participation in self-choice educational program (n = 281)

	**Adjusted ORs**	**95% CI Lower - Upper**	**p value**
Age (per year)	1.06	1.02 - 1.11	0.004
Gender			
Male	1.00	Reference group	
Female	5.84	2.67 - 12.79	< 0.001
Education			
> Junior high school	1.00	Reference group	
≤ Junior high school	1.18	0.63 - 2.21	0.606
Health Insurance			
Free or commercial	1.00	Reference group	
Basic medical care or none	1.06	0.57 - 1.97	0.850
Income (Chinese Yuan/month)			
Medium/high (> 3,000)	1.00	Reference group	
Low-income (≤ 3,000)	2.14	1.12 - 4.10	0.022

ORs, odds ratios; CI, confidence interval; Dependent variable, patients agreed to participate in self-choice educational program: participation = 1, non-participating = 0.

**Figure 2:**
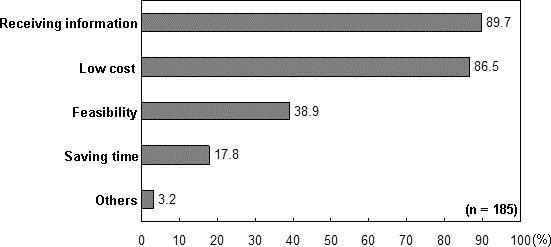
**Reasons for choosing self-choice educational program.** Bars represent the percentage of patients who provided the indicated answer. Patients could choose more than one answer.

When patients were asked why they preferred the self-choice educational program over CR, they gave the reasons as listed in Figure 2. The foremost reason was their desire to learn about the disease and how to reduce risk factors. Low costs were considered the second most important reason for their decision. Other reasons were its feasibility and time-saving nature.

Lastly, patients were asked what they wanted to learn from health professionals (Table 5). The majority of patients wanted to know how to exercise, manage risk factors, and gain knowledge about diseases, diet, and pharmacotherapy. Lifestyle changes and career advice were considered important information in less than half of the study patients. In addition, a minority of patients reported that they needed advice on strategies for coping with stress and depressed mood.

**Table 5:** Preferred information regarding secondary prevention among patients who agreed to participate in self-choice educational program

Relevant information*	**Patients (n = 185), n (%)**
Exercise	145 (78.4)
Modifying risk factors	127 (68.6)
Knowledge about the disease	119 (64.3)
Symptom management	107 (57.8)
Nutritional counseling	105 (56.8 )
Pharmacotherapy	98 (53.0)
Lifestyle changes	72 (38.9)
Career advice and returning to work	60 (32.4)
Management of depressed mood	45 (24.3)
Stress management	34 (18.4)

*Patients could give more than one choice.

There was no significant difference in scores of physical function and mental health among three groups at baseline. After 12 weeks from hospital discharge, there was an improvement in scores of physical function and mental health, with an increase of 79.7% and 58.6% in CR group, 27.6% and 22.4% in educational program group, and 18.8% and 11.2% in patients who did not participate in CR or educational program. These results showed that CR and self-choice educational program had a profound effect on physical function and mental health (Table 6, p < 0.001).

**Table 6:** Percent changes in scores of SF-36 Questionnaire after 12 weeks from hospital discharge in all study patients

**Variables**	**Refusal to attend CR (n = 281)**		**Agreed to attend CR (n = 47)**	**p value**
	**Agree to attend EP (n = 185)**	**Refusal to attend EP (n = 96)**		
Physical function, %	27.6 (18.2 - 41.4)	13.8 (6.0 - 26.8)	79.7 (58.5 - 108.7)	< 0.001
Mental health, %	22.4 (11.1 - 44.5)	11.2 (5.2 - 25.0)	58.6 (41.5 - 125.2)	< 0.001

Data are represented as the median (interquartile); SF-36, Short Form 36-Item Health Survey; CR, cardiac rehabilitation; EP, educational program; P value represents statistical difference among three groups.

## Discussion

Of the eligible patients who were offered a standard CR program, only a low proportion of patients chose this option. It is evident that there are many factors leading to poor participation rates for CR. Insurance coverage has been shown to be a major factor impacting participation in CR [11,24]. Patients without insurance are less likely to participate, which agrees with previous studies that report a lack of funding from insurance or other sources to impact attendance [16,24]. In agreement with this, very few people have free medical care or other commercial health insurance in mainland China [25]. Although the majority of the population are covered by the National Health Service in mainland China, the proportion of people who receive reimbursement for their medical treatment fees among those with medical insurance varies significantly by the type of medical insurance [25]. Moreover, the treatment costs for most patients are only partially reimbursed through basic medical care when they become ill and are admitted to the hospital. The average proportion of medical payments paid out-of-pocket is approximately 60% for the whole population, and government expenditures for health account for only 36% of the total health expenditure [4,26]. In addition, according to the reimbursement system, the National Health Service is unlikely to pay for a patient’s participation in a CR program after hospital discharge [5]. This further leads to a financial burden for most patients.

Socioeconomic status also reflects financial and material well-being, factors that may contribute to participation in CR. Similar to findings in industrialized countries [14,27,28], patients with higher income and education were more likely to attend CR. Given the association between cost and insurance coverage, lower educational and economic status may serve as markers for lack of insurance. In addition, patient characteristics such as age and gender have been reported to be significant predictors of participation in CR. In agreement with previous studies [10,12,13], older patients and women were less likely to attend CR. The reason for this disparity may be related to patients’ preferences, but it is even more likely that gaps in CR participation for these groups are due to financial strains, self-exercise, time conflicts, transportation problems, or a lack of family support. The most common reason for patients’ refusal to attend CR was that patients could not afford CR, which may be attributed to perceived insufficient health care resources and an under-developed reimbursement system. Understanding patient-reported reasons for their decision may further help understand the barriers associated with CR participation. Thus, a lack of emphasis, resources, and institutional support for such services are a few of the most important obstacles preventing greater participation in CR programs in mainland China.

The majority of patients in our study showed a clear preference for choosing the CR alternative, a self-choice educational program offered at minimal cost. Patients provided a wide range of reasons for their choices. Of these, receiving more information, the low cost, feasibility, and saving time were the most frequently given advantages of the self-choice educational programs. The strong desire for information among patients after acute cardiac events agrees with previous reports [29], and this indicates that the amount of information received during hospital stays are low. During a patient’s stay in the acute care, it is unlikely that physicians and nurses provide effective education about lifestyle modifications due to the short duration of the hospital stay as well as the high physical and emotional stress experienced by the patient during this phase of hospitalization. Additionally, because information may have been delivered at a time, patients were unable to completely understand some information by medical staffs and the need for information increases when patients are left on their own to handle their problems [30]. In this situation, the availability of self-choice educational programs at low cost, preferred by the majority of patients due to its affordability and feasibility, is a better opportunity to provide information about their disease, educate them about compliance with treatment regimens, and make informed decisions about lifestyle changes.

Women were more likely to prefer educational programs. Heart disease in women is characterized by a poorer prognosis, greater disability, and a higher rate of morbidity and early death after myocardial infarction compared with men [31]. Women often underestimate their risk for heart disease [32,33] and have longer pre-hospital delays than men [34]. After an acute cardiac event, women did not feel that they had received enough information from a health professional about treatment and preventive health behaviors and wanted a great deal of information about the management of their disease [29]. In accordance with a previous study [35], older patients wanted more information on disease management and prevention than younger and employed patients. The following explanations of these results are possible: 1) older age is associated with receiving less information during hospitalization [30]; 2) older patients may need information repeated due to age-related visual and hearing problems. In addition, lower-income patients wanted to attend educational programs, which was in line with recent evidence that low-socioeconomic status participants showed similar attendance and adherence to program guidelines as their higher-socioeconomic status counterparts [36].

The findings from this study will have significant implications for those seeking options for the management of CHD, particular for disadvantaged people with inadequate medical resources. Educational programs, as a simple and effective alternative, could provide sufficient information about the disease process and health promotion, disease prevention and risk education to those patients who are not able to access standard CR. However, a single education intervention seems to be insufficient as a means to obtain sustainable and meaningful benefits [37]. Thus, it should be emphasized that, wherever possible, standard CR should be used as part of the outpatient treatment plan following cardiac events based on evidence-based guidelines [38].

Despite the effectiveness of conventional centre-based CR programs, participation rates are low [15,39] and the majority of patients requiring CR are missing out on evidence-based health benefits of lifestyle interventions. Little research has been conducted on improving health outcomes for the majority of cardiac patients who do not attend CR. This study addresses these gaps in public health practice by testing an alternative delivery mode for CR. While this study leads to conclusions specific to mainland China, it has implications for other countries as well. More research is needed to explore possible strategies to overcome these barriers to participation in CR.

### Limitations

Our study has several limitations, particularly with regards to how we measured CR or self-choice educational program attendance. Attendance at CR or self-choice educational program was defined as having attended at least one session. We did not differentiate between patients who completed all sessions and patients who only ever attended one session. This precluded investigations associating between patient preferences and non-completion of the CR or self-choice educational program. The National Service Framework set the target at 85% for the number of eligible patients invited to join rehabilitation programs [40]. This study reports only on the numbers participating in rehabilitation programs, so it can only indirectly reflect the failure to meet this target. Although participation rate probably included a few who joined the program but dropped out too soon to gain the real benefit, the one of goals of this study were to examine the obstacles to CR and patient’ wishes to attend alternative secondary preventive measures. In fact, only few patients dropped out of CR (2.1%) or of self-choice educational programs (3.8%) according latest data. The second main limitation pertains to measurement. Although the validity of questionnaire regarding preferences for CR or a self-choice educational program was not verified, the potential for social desirability biases in participant responses could be ruled out. Patients were asked about their preferences and reasons for their decisions prior to discharge. This enabled the exploration of patient preferences at a time when patients were likely to be making decisions about their own secondary preventive measures.

There were also other limitations. First, our study only considered a sample from one localized geographical location. Thus, it may not be possible to generalize our results to those living in other regions of China. Secondly, we had a relatively small study population, and our participants were recruited from a single center with a stable population. Finally, there is a lack of long-term follow-up data. We are not able to assess the extent to which participation in either secondary prevention program sponsored by our center affected the prognosis of these patients.

## Conclusions

There were many barriers at the levels of the patient, the health system, and society that prevented eligible patients from accessing CR services. It is essential to provide a flexible model for CR delivery that is suitable for local conditions and meets the needs of patients. The majority of patients expressed a strong desire for self-choice educational programs to obtain more information about disease and health promotion even after hospital discharge, especially among female, older, and low-income patients. Findings from this study may be used to guide health professionals in providing or offering effective, safe, convenient and culturally relevant programs for CHD secondary prevention.

## Abbreviations

CHD: Coronary heart disease; CR: cardiac rehabilitation; PCI: Percutaneous coronary intervention; ACS: Acute coronary syndrome; STEMI: ST segment elevated myocardial infarction; NSTEMI: Non-ST segment elevated myocardial infarction; CABG: Coronary artery bypass graft; ANOVA: One way analysis of variance; SF-36: Short form 36-item health survey.

## Competing interest

The authors declare that they have no competing interest.

## Authors’ contributions

HJ was the principal investigator, involved in designing the study, analyzing the data, and writing the manuscript. QW and LC contributed to data collecting. HJ, QS, YZ and JW were responsible for exercise training and educational programs. NFL and GSM provided expertise in the research design and plan coordination. All authors read and approved the final manuscript.

## Pre-publication history

The pre-publication history for this paper can be accessed here:

http://www.biomedcentral.com/1471-2261/14/20/prepub
